# Genomic variant-driven prediction of azole resistance in *Aspergillus fumigatus* using GWAS and machine learning

**DOI:** 10.3389/fmicb.2026.1891518

**Published:** 2026-07-07

**Authors:** Dingchen Li, Xinkai Yue, Wenjuan Hu, Hanying Zhong, Fangyan Chen, Jingya Zhao, Luyao Cao, Xia Chen, Li Han

**Affiliations:** 1 Department for Disinfection and Infection Control, Chinese PLA Center for Disease Control and Prevention, Beijing, China; 2 Department of Information, Chinese PLA Center for Disease Control and Prevention, Beijing, China; 3 School of Mathematics and Statistics, Xi’an Jiaotong University, Xi’an, Shaanxi, China; 4 School of Mathematics and Statistics, Shaanxi Normal University, Xi’an, China

**Keywords:** *Aspergillus fumigatus*, azole resistance, GWAS, machine learning, SNP calling

## Abstract

Azole resistance in *Aspergillus fumigatus*, a major cause of invasive aspergillosis, threatens public health. Known drivers include *cyp51A/B* mutations (e.g., TR34/L98H and TR46/Y121F/T289A), yet existing studies have largely focused on clinical isolates and known genetic determinants, leaving gaps in understanding broader genomic contributions. This study aimed to develop a machine learning–based framework to overcome limitations of traditional GWAS and identify novel resistance loci beyond *cyp51A*. A global collection of 590 *A. fumigatus* strains was analyzed, including whole-genome sequencing (WGS) data from 15 countries and resistance phenotypes using CLSI/EUCAST guidelines. Phylogenetic analysis revealed four clades without geographic clustering. Clade III harbored the highest proportion of resistant strains (ITR: 51.89%, POS: 50.48%, VOR: 38.68%), predominantly linked to *cyp51A* tandem repeats. In contrast, Clade IV strains frequently carried point mutations but showed lower resistance rates. GWAS was performed using PLINK and GAPIT frameworks, and 7,098 high confidence SNPs were selected for ML modeling. Ten classifiers were evaluated using repeated random 80:20 train-test splits, with five-fold cross-validation used for RFECV-based feature selection and model tuning where applicable. RF and XGBoost achieved superior performance, with mean AUCs > 95% and accuracy > 88% across all azoles. Penalized logistic regression outperformed SVM and AdaBoost. Decision trees exhibited the lowest accuracy. The SNP SCM000172.1_1781459 was identified as a key predictor for all three azoles. Cross-resistance analysis revealed significant overlap between ITR and POS resistance loci, whereas VOR-associated loci were distinct, suggesting divergent mechanisms. The findings provide actionable insights for resistance surveillance, antifungal development, and tailored treatment strategies.

## 1 Introduction

### 1.1 Background

*Aspergillus fumigatus* (*A. fumigatus*), a saprophytic filamentous fungus prevalent in soil rich in decaying organic matter, is the principal pathogenic species implicated in aspergillosis, contributing to approximately 90% of clinical manifestations (Perfect et al., 2001). The incidence of aspergillosis has been progressively increasing, largely due to the augmented use of invasive surgical procedures, immunosuppressive therapies, and organ transplantation, resulting in over 200,000 life-threatening cases reported worldwide annually (Brown et al., 2012). Due to its spore dispersal characteristics, *A. fumigatus*, can serve as a carrier for fungal biological warfare agents and holds significant potential in military medicine. Significantly, throughout the COVID-19 pandemic, an increasing body of literature has reported on coinfections involving the novel coronavirus and *A. fumigatus* (Lai and Yu, 2021; Borman et al., 2020). Widespread clinical and environmental azole exposure has contributed to the emergence and detection of azole-resistant *A. fumigatus* isolates in multiple regions (Sewell et al., 2019). Therefore, elucidating genomic mechanisms associated with azole resistance is important for infection-control strategies and antifungal therapeutic development, while functional validation remains necessary to establish causal mechanisms (Harrison et al., 2026).

Since the 1990s, azole-resistant isolates of *A. fumigatus* have been progressively identified in various countries, including the United Kingdom, Netherlands, Norway, Spain, Belgium, Denmark, France, Germany, India, Iran, Portugal, Brazil, and the Czech Republic. In China, the initial detection of itraconazole-resistant *A. fumigatus* occurred in Beijing in 2005, and subsequent reports have documented the presence of azole-resistant strains in additional regions such as Hangzhou, Fujian, Shanghai, Nanjing, and Taiwan (Snelders et al., 2008). Globally, there has been an increasing trend in the detection rates of azole-resistant *A. fumigatus* strains. In the United Kingdom, the prevalence of azole-resistant strains was reported to be between 5 and 7% in 2004. However, this figure rose to 20% by 2006, and by the period of 2008–2009, the detection rate had escalated to 28%. Similarly, the incidence of azole-resistant *A. fumigatus* (Seyedmousavi et al., 2013; Bueid et al., 2010; Gonçalves et al., 2013), strains has been increasing in other countries, such as Netherlands and the United States (Alastruey-Izquierdo et al., 2013). Consequently, azole resistance in *A. fumigatus* has merged as a significant global concern.

### 1.2 Current research status on drug resistance mechanisms in *Aspergillus fumigatus*

In *A. fumigatus*, two homologous genes, *cyp51A* and *cyp51B*, encode the Cyp51 protein. The predominant mechanism of underlying azole resistance in clinical strains is associated with alterations in Cyp51, primarily attributed to Cyp51, mainly due to point mutations and the overexpression of *cyp51A*. In contrast, *cyp51B* assumes a functional role under specific conditions (Hu et al., 2007). Notably, *cyp51B* may exhibit elevated or constitutive overexpression in clinical azole-resistant strains (Mellado et al., 2005). Experimental investigations have revealed that point mutations at various sites within *cyp51A*—such as G448S, G54A, G54W, P216I, and M220V/K/T—contribute to azole resistance (Blatzer et al., 2011). These sites constitute essential regions adjacent to the heme core of the Cyp51A protein. Mutations within these areas can modify the structure and conformation of Cyp51A, thereby impeding the effective binding of azole drugs and leading to drug resistance through a mechanism known as “target escape.” The overexpression of *cyp51A* is frequently associated with the insertion of tandem repeat (TR) in its promoter region, exemplified by TR34 and TR46. These tandem repeats often co-occur with specific point mutations in *cyp51A*, with TR34/L98H and TR46/Y121F/T289A being prevalent combinations. The integration of these TR sequences not only augments the binding affinity of transcriptional activators, thereby elevating the expression of the *cyp51A* gene, but also impedes the binding of transcriptional repressors, consequently facilitating *cyp51A* overexpression. In addition to TR insertions, *cyp51A* expression is modulated by transcriptional regulators such as SrbA, AtrR, and HapE. The absence of SrbA results in diminished *cyp51A* expression, rendering *A. fumigatus* less resistant to azole antifungal agents (Paul et al., 2019). AtrR, another zinc-finger protein, is responsible for the regulation of both *cyp51A* and *cyp51B* gene. The deletion of AtrR not only diminishes the expression of these genes but also attenuates the virulence of the strain (Hagiwara et al., 2017). Moreover, a P88L mutation in the CCAAT-binding transcription factor HapE leads to overexpression of cyp51A and azole resistance. Additionally, cytochrome CybE has been identified as a regulator of *cyp51A* transcription, and its deletion results in compensatory upregulation of cyp51A and the accumulation of the ergosterol precursor eburicol (Camps et al., 2012; Misslinger et al., 2017).

Triazole-resistant strains have been extensively documented and characterized across numerous countries globally. Most of these investigations have concentrated on the prevalence of resistant strains within clinical settings. Furthermore, most analyses of the mechanisms underlying triazole resistance have emphasized mutations in the *cyp51A* gene, which encodes the target enzyme for triazoles (Verweij et al., 2016). Among clinical resistant strains that emerge during aspergillosis treatment, the most prevalent mutations in *cyp51A* occur at amino acid sites G54, G138, M220, and G448 (Lestrade et al., 2019; Howard et al., 2009). Concurrently, the predominant triazole drug-resistant mutations identified within the global *A. fumigatus* population are TR34/L98H and TR46/Y121F/T289A, with a significant number of these resistant strains originating from environmental sources (Chowdhary et al., 2013).

In addition to cyp51A-mediated mechanisms, azole resistance can involve non-cyp51A pathways, including altered expression or function of drug transporters, transcriptional regulators, sterol-biosynthesis genes outside cyp51A, and stress-response pathways. These mechanisms may contribute to resistance phenotypes that cannot be fully explained by known cyp51A substitutions or promoter tandem repeats. Structural variants and copy number variations may also contribute to azole resistance but are not fully captured by SNP-focused analyses. Promoter tandem repeats, gene duplications, copy-number changes, ortholog variation, or other large-scale variants can affect gene expression and drug susceptibility (Horta et al., 2022). Therefore, the SNP-based design of the present study should be interpreted as prioritizing single-nucleotide candidate markers, while larger structural variants remain an important direction for future work.

### 1.3 Advancements in fungal GWAS (especially *Aspergillus fumigatus*)

Microbial genome-wide association studies (GWAS) have emerged as a potent methodology for elucidating the relationships between genetic variation and microbial phenotypes (Denning et al., 2013). The cornerstone of GWAS lies in the exhaustive and methodical examination of genome-wide data, presenting an innovative methodology for exploring the genetic mechanisms underlying phenotypic traits associated with drug resistance in pathogenic bacteria. With advancements in technology, GWAS has facilitated the identification of numerous pathogenic bacteria, elucidating the genetic determinants associated with essential phenotypic characteristics. However, research concentrating on invasive fungi remains scarce, primarily due to the intricate nature of their genomes and genetic materials.

Nevertheless, some successful GWAS applications have been undertaken with the aim of identifying novel genomic markers associated with antifungal drug resistance. In this context, numerous studies have concentrated on investigating azole resistance in plant fungal pathogens (Mohd-Assaad et al., 2016). For instance, Etienne *et al*. performed a comprehensive genome-wide single nucleotide polymorphism (SNP) analysis on 190 *A. fumigatus* strains from the USA, Netherlands, India, and the UK, which uncovered the presence of two distinct populations (A and B) within the USA (Talas et al., 2016). The resistant mutant strain TR34/L98H was identified as forming a distinct evolutionary clade (clade A), potentially sharing a common origin with resistant strains from other geographical regions. Notably, 6% of the resistant strains were found to be distributed across both clades, suggesting the possibility of genetic recombination (Etienne et al., 2021; Losada et al., 2015). Additionally, Losada *et al*. conducted a comprehensive genomic analysis of the progeny of resistant strains using drug sensitivity assays and mating passaging, which led to the identification of five genes associated with azole resistance in *M. fumigatus*. This indicates that the mechanisms of resistance encompass factors beyond those linked to CYP51A. In a comprehensive whole-genome sequencing analysis of 196 *A. fumigatus* strains from 11 countries, Fan *et al.* identified three genetic clusters within a phylogenetic tree (Fan et al., 2020). They observed that 90% of the resistant strains were concentrated within a single cluster. A total of over 60 SNPs significantly associated with resistance to amphotericin B (AMB) were identified. Recently, Zhao and colleagues performed a GWAS to investigate itraconazole susceptibility in non-resistant clinical isolates from Japan (Zhao et al., 2021). Concurrently, Yuying Fan *et al*. undertook a GWAS to examine resistance to itraconazole and voriconazole in 195 *A. fumigatus* strains utilizing a globally available genomic dataset (Fan et al., 2021).

### 1.4 The current state of machine learning technology applications

Machine learning-based predictive models have demonstrated the capability to explore numerous potential associations between genetic variants, thereby providing a valuable tool for researchers in this field. This technology has been incorporated into various fields, encompassing data generation, subsequent analysis, and the mining of knowledge therefrom. As a result of the swift advancements in machine learning in recent years, a growing number of researchers are utilizing these methodologies for phenotypic-genotypic association studies. Recently, several studies have employed machine learning algorithms to predicted microbial drug resistance (Deo, 2015).

For instance, Zhang *et al*. utilized logistic regression and random forest models to identify and predict seven novel genes associated with drug resistance in *Mycobacterium tuberculosis* (Zhang et al., 2013). Conversely Yang *et al*. adopted a more comprehensive array of machine learning classifiers, including logistic regression (LR) with an L2 penalty, support vector machines (SVM), random forests, decision trees (RF), gradient boosting (GB), and AdaBoost, among others. Additionally, they conducted comparative analyses of classification models for eight antimicrobial agents and predicted novel mutation sites associated with drug resistance in *Mycobacterium tuberculosis* (Yang et al., 2018). Moreover, machine learning methodologies have been integrated with genome-wide association studies to investigate the relationships among genetic variants. For example, Maisem *et al*. predicted the virulence of bacterial MRSA using the genome sequence of the bacteria (Laabei et al., 2014). Their study employed the random forest algorithm for regression analysis and prediction, culminating in a model that demonstrated high accuracy in predicting the toxicity of the isolates. Meanwhile, Zhang *et al*. employed machine learning techniques, specifically support vector machine regression and regularization methods such as lasso and ridge regression, in combination with genome-wide SNP markers for the genomic prediction of fall dormancy (FD) (Zhang et al., 2023). Their findings indicated that utilizing linear kernel SVM regression with the top 3,000 markers associated with GWAS achieved the highest FD prediction accuracy, reaching 64.1%.

Recent studies have increasingly combined genome-wide variants with machine-learning methods for antimicrobial-resistance prediction, and related machine-learning approaches have also been applied to fungal genomic traits and antifungal-resistance signatures (Horta et al., 2022; Dort et al., 2023). Accordingly, the present study does not claim that the general combination of GWAS and machine learning is unprecedented. Its specific contribution is the use of a GWAS-informed feature-reduction strategy, population-structure-aware association analysis, repeated model evaluation, and functional annotation to prioritize candidate azole-resistance markers in *A. fumigatus*.

### 1.5 Research objectives

In this study, *A. fumigatus* strains were collected from diverse countries and regions, and their whole genome sequencing data were analyzed to construct phylogenetic relationships and evaluate population structure. The high dimensionality of the genetic data, coupled with the prevalence of missing values and the presence of irrelevant and redundant information, posed significant challenges for the direct application of raw data in machine learning model development. To overcome these challenges, we implemented multiple GWAS models as a feature-screening step for azole-resistance prediction in *A. fumigatus*. Significant loci identified through GWAS analysis were selected as feature variables for machine learning modeling, thereby, effectively reducing data dimensionality and simplifying the construction of the model.

We utilized multiple machine learning classifiers to develop a predictive model for azole resistance in *A. fumigatus* and conducted a comprehensive evaluation and comparison of the classification performance of each classifier. By integrating recursive feature elimination with cross-validation (RFECV) and penalized machine-learning approaches, we ranked candidate mutation sites associated with azole resistance in *A. fumigatus.*

The primary aims of this study were to identify genetic variants associated with triazole resistance, with a particular emphasis on novel mutations not related to *cyp51A*, and to perform phylogenetic analyses of 590 strains to evaluate resistance to itraconazole, posaconazole, and voriconazole.

## 2 Materials and methods

### 2.1 Data and pre-processing

This investigation utilized 590 strains incorporating whole-genome sequencing (WGS) data for those with available resistance information including 107 *A. fumigatus* strains sequenced by the Center for Disease Control and Prevention (CDC) of the Chinese People’s Liberation Army. The remaining 483 isolates obtained from public databases such as NCBI and EBI. Based on Supplementary Table S1, geographic metadata were available for 551 isolates: Germany (*n* = 253), China (*n* = 91), Netherlands (*n* = 40), Japan (*n* = 38), USA (*n* = 37), United Kingdom (*n* = 30), Spain (*n* = 24), India (*n* = 12), Canada (*n* = 12), Portugal (*n* = 8), ISS (*n* = 2), Ireland (*n* = 1), France (*n* = 1), Peru (*n* = 1), and Singapore (*n* = 1). The 39 isolates without geographic metadata were included in the analysis because their sequencing data and resistance phenotypes met the quality-control criteria.

Currently, two recognized standard protocols for antifungal susceptibility testing are in use. The first protocol is the CLSI M38-A2 guideline, formulated by the Clinical and Laboratory Standards Institute (CLSI) in the United States. The second protocol is the EDef 9.3 standard method, issued by the European Committee on Antimicrobial Susceptibility Testing (EUCAST) (Laabei et al., 2014; Clinical and Laboratory Standards Institute, 2008). These guidelines establish standardized procedures for assessing the efficacy of antifungal agents against a range of fungal pathogens, thereby ensuring consistent and reliable outcomes in clinical laboratory settings.

**Figure 1:**
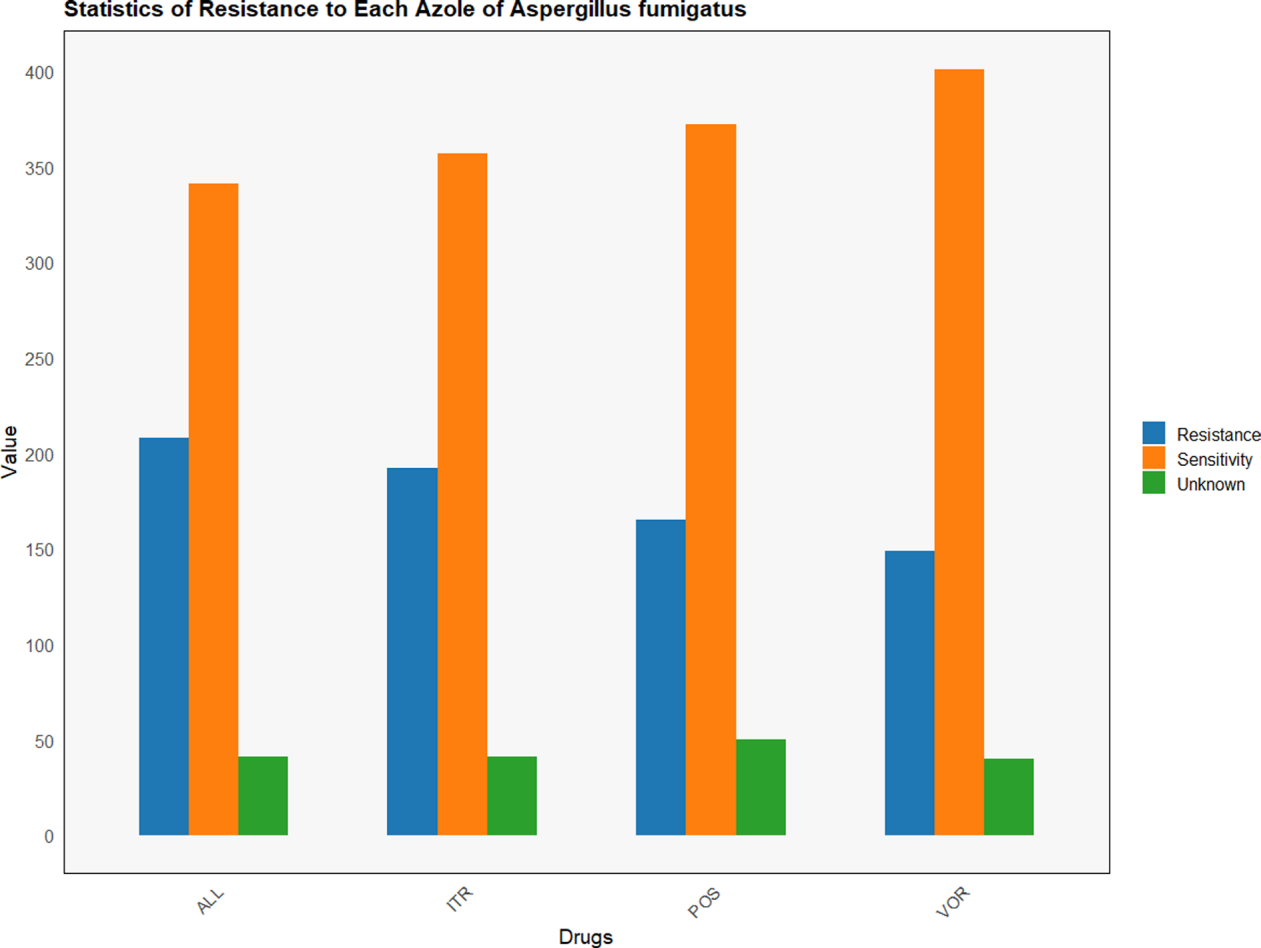
Statistics of resistance to each azole of *A. fumigatus*.

The *A. fumigatus* WGS data used in this study were generated using the Illumina sequencing technology platform. The study examined 590 strains of *A. fumigatus*, with a focus on resistance to the antifungal agents voriconazole (VOR), posaconazole (POS) and itraconazole (ITR). Resistance/susceptibility categories were harmonized into resistant, susceptible, and unknown classes according to the classification reported by the source study or laboratory metadata, which were based on CLSI M38-A2 or EUCAST methods where available. Isolates with unknown phenotype for a given azole were excluded only from that drug-specific model. The drug resistance profiles for each strain are detailed in Table 1 and Figure 1.

**Table 1:** Statistical table of resistance to three azoles in *A. fumigatus*.

Drugs	VOR	POS	ITR
Resistance	149	165	192
Susceptible	401	372	357
Unknown	40	53	41

### 2.2 Whole genome sequencing and data preprocessing

The data for the strains selected in this study were sourced from three primary origins. A portion of the data was retrieved from the publicly accessible databases, specifically the EBI and the NCBI. Additional data were derived from the aspergillus strains referenced within the article. The remaining data were obtained from the Chinese PLA Center for Disease Control and Prevention (PLACDC), which provided a proprietary collection of newly sequenced strains, comprising 590 isolates. WGS data for these 590 *A. fumigatus* strains are available in the Supplementary material. All strains underwent sequencing via the Illumina Sequencing Technology platform, with the sequencing data presented in paired-end WGS format in FASTQ file. Antifungal susceptibility testing was performed on these strains, and based on the results, strains were classified as resistant or sensitive to triazoles in accordance with the Clinical and Laboratory Standards Institute (CLSI) guidelines. The resistance profile of these strains included itraconazole, voriconazole, posaconazole and amphotericin B (AmB). This study specifically concentrated on strains regarding azole resistance.

### 2.3 Variant calling

The initial sequencing data obtained from the strains were in FASTQ format, containing both biological sequence information and base-quality metrics for each sample. Following data quality assessment, quality control, sequence alignment, PCR duplicate marking, base-quality recalibration, variant calling, and quality filtering, a VCF (Variant Call Format) file was generated. Raw reads were assessed using FastQC, trimmed using Trimmomatic (Bolger et al., 2014), and aligned to the *A. fumigatus* Af293 reference genome using BWA (Li and Durbin, 2009). Alignment files were sorted using Samtools (Li et al., 2009), and variant calling and filtering were performed using GATK v4.1.4.1 (McKenna et al., 2010). The reference genome was *A. fumigatus* Af293 assembly ASM265v1, RefSeq accession GCF_000002655.1/GenBank accession GCA_000002655.1. GATK hard filtering used thresholds of QUAL < 30.0, QD < 2.0, FS > 60.0, MQ < 40.0, DP < 5, SOR > 3.0, HaplotypeScore > 13.0, MappingQualityRankSum <−12.5, and ReadPosRankSum <−8.0. A total of 689,079 loci were identified and used as the initial SNP reference set. Subsequent variant filtering was conducted using VCFtools to exclude indels, low-quality variants (Danecek et al., 2011), variants with insufficient call rate, multiallelic sites, and non-biallelic variants, retaining only biallelic SNPs for subsequent analyses.

To mitigate the effects of linkage disequilibrium (LD) within the linkage imbalance interval, SNP markers exhibiting linkage were filtered using a sliding window approach with PLINK 1.90 beta, employing the parameter indep-pairwise 100 50 0.2 (Purcell et al., 2007). This procedure scans 100-SNP windows, shifts the window by 50 SNPs at each step, and removes one SNP from marker pairs with r2 greater than 0.2. The final number of filtered loci was 527,231.

Variants were annotated using SnpEff version 5.0, in conjunction with the Af293 reference genome annotation (Cingolani et al., 2012). These files were prepared for phylogenetic analysis and machine learning-based resistance analysis.

To facilitate subsequent analyses, Tassel software was employed to convert the VCF format files into HapMap format (Bradbury et al., 2007).

### 2.4 Phylogenetic analysis

The missing genotypes were estimated using Beagle software (Browning and Browning, 2007). To determine the evolutionary relationships among the 590 samples, the nucleotide sequences of the SNP loci for each sample were concatenated. Phylogenetic neighbor-joining (NJ) trees were constructed using MEGA-X, with the bootstrap test conducted 500 times for validation (Kumar et al., 2018). The resulting phylogenetic tree was subsequently annotated using the iTOL online tool^1^ (Letunic and Bork, 2021).

**Figure 2:**
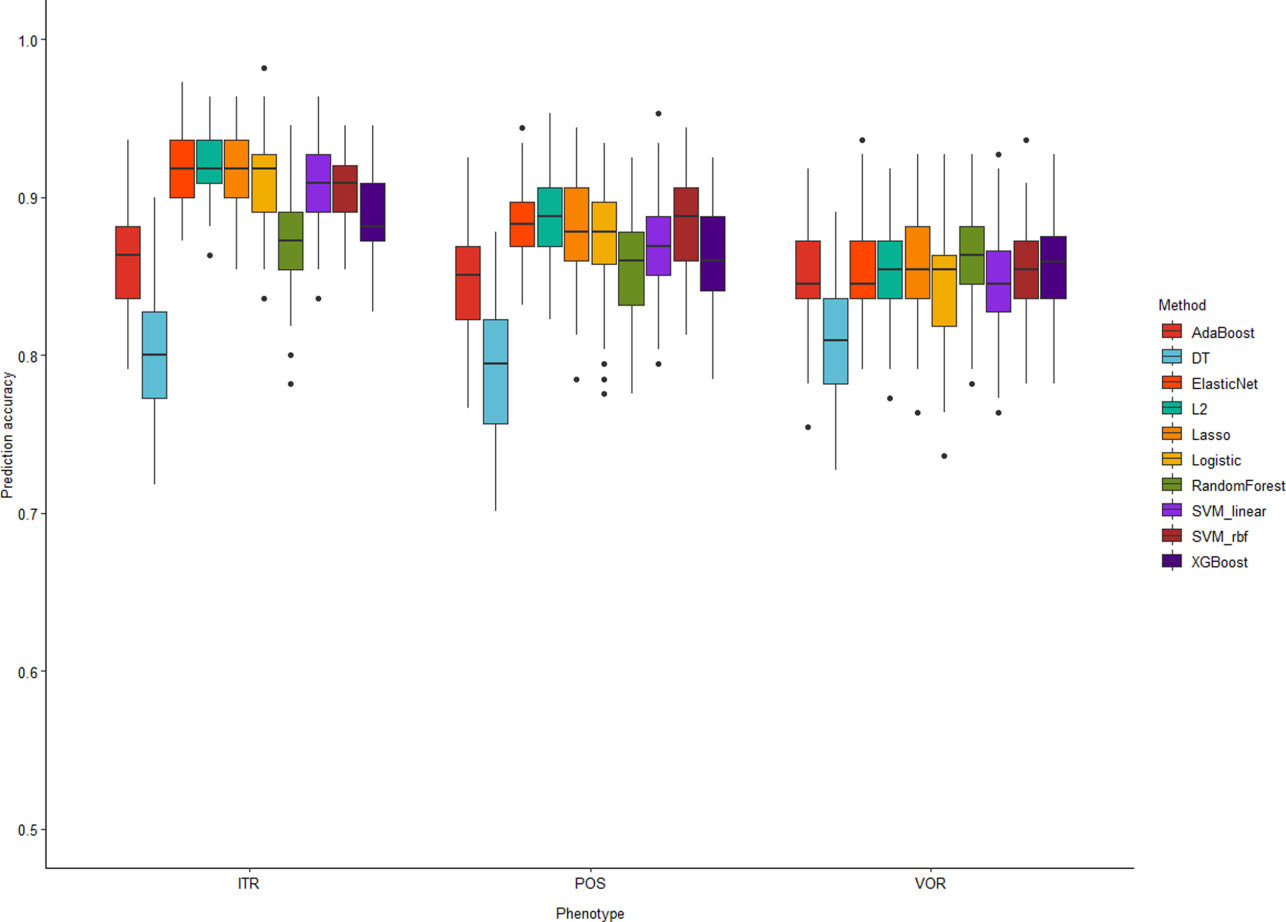
Neighbor-joining phylogenetic tree detailing the strain characteristics. Branches with a red dot represent those with over 50% bootstrap support, derived from 500 bootstrap iterations. The inner-most circle denotes the clade affiliation of strains with strain names corresponding to those listed in Supplementary Table S1. The second inner-most circle represents the country or origin for each strain with distinct colors representing different countries as shown in the left “Country” panel. The colored five-pointed star in the middle of the image indicates whether the strain has a known resistance-associated mutation. The different colors indicate different known mutation sites as shown in the left “Common mutation” panel. The term “null” denotes the absence of a mutation at a specific site. The three colored bands in the center (blue, red, and green) represent the resistance of the strains to the three drugs itraconazole ITR, VOR and POS, respectively. The outermost circle indicates mutations in the cyp51A gene.

The phylogenetic NJ tree is divided into four distinct clades originating from the root, as depicted in Figure 2: Clade I(rose), Clade II(mauve), Clade III(brown) and Clade IV(cyan). Red circles on the branches denote the Bootstrap Support Proportion during the construction of the tree. Among the strains analyzed, one strain was classified in Clade I, 28 strains were in Clade II, 107 strains in Clade III and 455 strains in Clade IV.

The nodes are annotated with the nomenclature of each strain, and the information pertinent to each sample is represented by three concentric circles centered on the tree. The innermost circle displays the geographical origin of the strain, with different colors indicating the country or region of origin. Geographically, Clade II comprises strains from five countries, including one strain each from Canada and Peru, one strain from Portugal, three strains from Spain, and 13 strains from Germany. Clade III consists of strains from eight countries, including four strains from North America, 69 from Europe, seven from India, and 24 from China. Clade IV encompasses strains identified in the following 13 countries: Canada (*n* = 10), China (*n* = 82), France (*n* = 1), Germany (*n* = 196), India (*n* = 5), Ireland (*n* = 1), Japan (*n* = 38), Netherlands (*n* = 17), Portugal (*n* = 7), Singapore (*n* = 1), Spain (*n* = 18), UK (*n* = 16), USA (*n* = 34). In addition, two strains were isolated from the international Space Station (ISS), and one strain from Clade I originated from China.

The subsequent section offers a comprehensive analysis of the sub-clusters within the phylogenetic tree for each country or region concerning *A. fumigatus*, as detailed in Supplementary Table S1.

It is evident that there is no clustering of strains based on geographic location, indicating that the evolution of *A. fumigatus* is not geographically constrained. No clear clustering of isolates by geographic origin was observed in the phylogenetic tree, suggesting limited geographic segregation within the sampled *A. fumigatus* population. This pattern is consistent with the high dispersal capacity reported in previous studies (Abdolrasouli et al., 2015).

As illustrated in Figure 2, the three colored annular bands (blue, red and green) denote the resistance of the strain to the antifungal drugs itraconazole (ITR), voriconazole (VOR) and posaconazole (POS), respectively. The corresponding blue, red and green regions correspond to resistance of this *A. fumigatus* strain to the specified antifungal drug. Whereas, the yellow region denotes sensitivity (non-resistance) to the same drug. The white region represents an indeterminate resistance status for the strain. At the clade level, the findings revealed that the percentage of itraconazole-resistant strains was 100% for Clade I, 3.7% for Clade II, 51.89% for Clade III, and 9.86% for Clade IV. For voriconazole, the proportions of resistant strains were 100% for Clade I, 3.7% for Clade II, 38.68% for Clade III, and 11.51% for Clade IV. In the case of posaconazole, the proportion of resistant strains was 100% for Clade I, 3.7% for Clade II, 50.48% for Clade III and 9.38% for Clade IV.

It can be observed that strains resistant to itraconazole are almost invariably resistant to posaconazole as well. Specifically, *A. fumigatus* samples demonstrate extensive multidrug resistance to both antifungal agents. However, resistance to voriconazole does not exhibit a clear correlation with the resistance profiles of the other two drugs. It is plausible that a substantial number of duplicated sites may confer resistance to itraconazole in *A. fumigatus*, while simultaneously inducing resistance to posaconazole without affecting resistance to voriconazole, a hypothesis corroborated by substantiated in subsequent analyses.

As illustrated in Figure 2, the central-colored pentagram in the diagram indicates whether the strain harbors a mutation associated with drug resistance. The outermost layer categorizes the mutation status of the cyp51A gene as follows: “unknown”, indicating unavailable information; “point_mutation”, denoting the presence of a point mutation in the cyp51A gene; “TR” (tandem repeat), signifying the occurrence of a tandem repeat in the cyp51A gene; and “None”, indicating the absence a mutation. Clade II predominantly comprises samples with point_mutations; Clade III primarily consists of TR and None classes; Clade IV encompasses a diverse range of classes, making it difficult distinguish, within which the proportion of point_mutation is 44.84%, the proportion of TR is 10.33%, and the proportion of None is 21.76%.

Supplementary Table S1 provides detailed information on the 590 strains and Clade divisions.

### 2.5 Genome-wide association study

The initial GWAS analyzes were conducted utilizing PLINK 1.9 beta, employing chi-square tests and logistic regression models deemed suitable for the binary traits under examination. Principal component analysis (PCA) was performed using PLINK, and the resulting principal components were incorporated as covariates in PLINK logistic regression. In the logistic regression (LR) model, ten principal component vectors were incorporated as covariates. Furthermore, the GAPIT framework was employed for GWAS analysis, applying its six in-built models: GLM, MLM, CMLM, MLMM, BLINK, and SUPER (Wang et al., 2014; Lipka et al., 2012). GAPIT also generated a Zhang kinship matrix from genotype data. Population structure was summarized by the first five PCs selected from scree-plot analysis, and genetic relatedness was further accounted for using kinship-aware mixed models, particularly MLM and MLMM. Although these strategies reduce confounding from population structure and clonal relatedness, residual confounding cannot be fully excluded.

**Figure 3:**
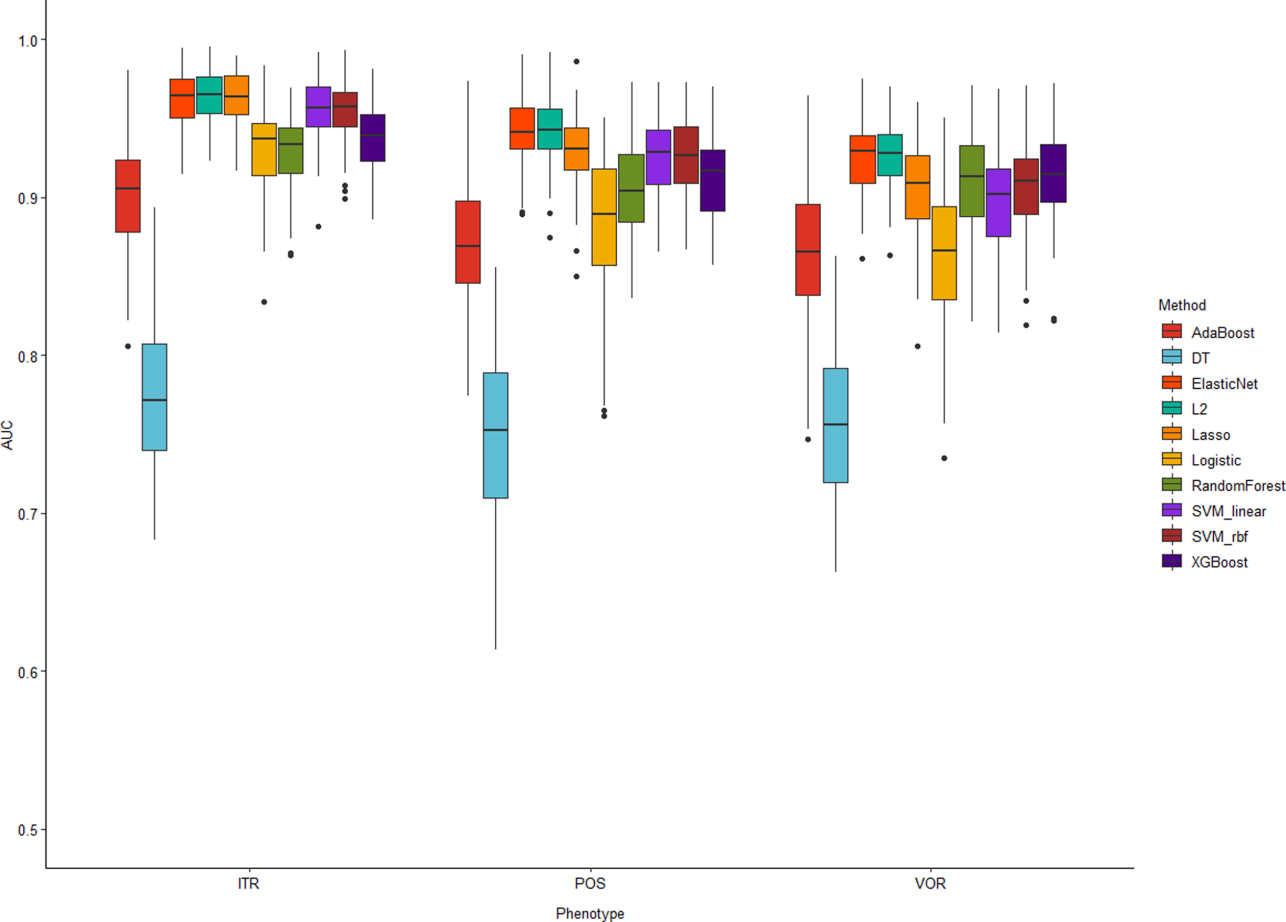
Quantile-quantile plot of genome-wide SNPs associated with *A. fumigatus* triazole resistance in different models.

**Figure 4:**
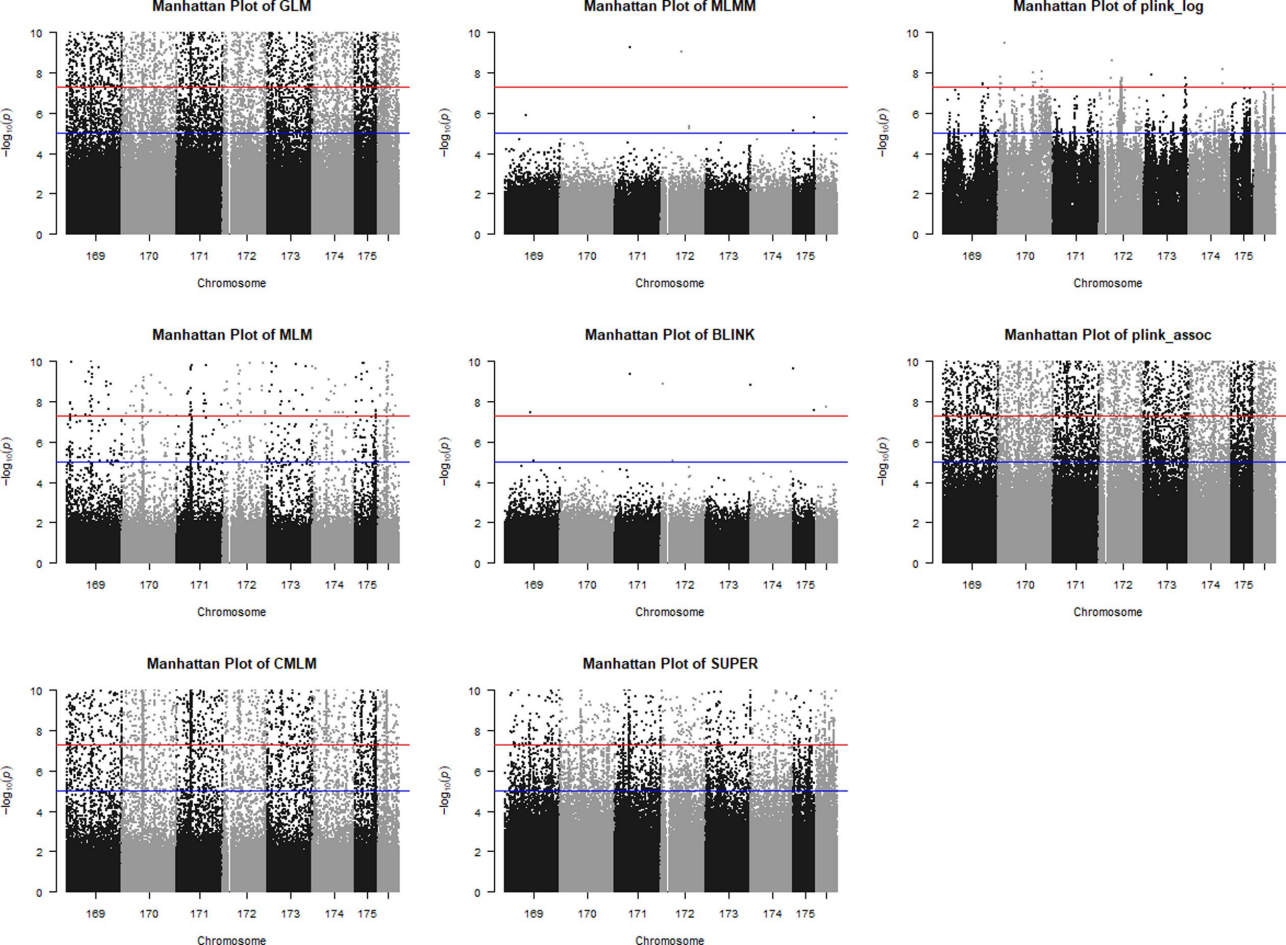
The Manhattan plot depicting genome-wide associations with triazole resistance in *A. fumigatus*. The different models are labeled at the top of each subfigure. The plot features chromosome positions on the X -axis and the −log_10_(*p*-value) on the Y -axis. Red dashed line and blue dashed line significance thresholds (*p*-value = 10^−7^ and 10^−5^ respectively).

Given the absence of systematic GWAS investigations in previous studies on *A. fumigatus* resistance, the most appropriate models for exploring the association between SNPs and resistance phenotypes remain undetermined. In this study, we performed GWAS analyses using two distinct software programs. The results of our analyses are summarized in Figures 3, 4.

### 2.6 Machine learning algorithms

The initial phase in constructing a machine learning classifier model involves the selecting the appropriate dataset. The GWAS conducted in the preceding phase was examined, revealing that three models exhibited relatively robust performance. However, the incidence of false positives remained high. Consequently, further screening of features at these significant loci is necessary to minimize false-positive loci and enhance the accuracy of significant correlation loci. It was thus decided to employ a machine learning approach for variable screening.

To reduce the risk of overfitting caused by the high dimensionality, we used GWAS-informed feature reduction before model construction. In addition, regularized logistic models, RFECV feature selection, and repeated internal train-test evaluation were applied to improve model stability. Nevertheless, because no independent external validation cohort was available, the reported performance should be interpreted as an internal estimate of predictive ability.

A *p*-value threshold of 0.01 was used as a permissive feature-screening threshold to generate candidate SNPs for downstream machine-learning analysis, rather than as a definitive genome-wide significance threshold. Across azole phenotypes and GWAS approaches, the MLMM model identified 4,624 SNP loci with significantly associated with *A. fumigatus* resistance. The BLINK model detected 3,527 candidate SNP features, while the logistic regression model in PLINK identified 34,046 candidate SNP features. The final set of 7,068 SNPs was not a strict intersection of these three models; instead, it represented a merged, non-redundant candidate SNP set obtained by combining SNPs across GWAS models and azole phenotypes followed by de-duplication. These SNPs are interpreted as candidates for model construction rather than confirmed causal loci.

Through a comprehensive review of existing literature, we identified a set of genes previously reported to be potentially associated with triazole resistance in *A. fumigatus*. These genes may include determinants of resistance to this drug. Consequently, these loci were integrated with the significant SNPs identified earlier, culminating in a total of 7,098 SNP loci for the development of a machine learning classifier model.

The SNP locus data were encoded into a 0-1-2 matrix format, and phenotypic data were encoded as binary variables, with 1 representing resistance and 0 representing susceptibility. Samples with unknown resistance status for a specific azole were excluded from the corresponding drug-specific analysis. Because resistant isolates were fewer than susceptible isolates for each azole phenotype, AUC was used as the primary performance metric, and ACC was interpreted together with class-wise performance metrics. This process resulted in a dataset suitable for machine learning classification modeling. The resistance phenotype of *A. fumigatus* to three different azoles was utilized for evaluation across various models.

An algorithm was developed using Python version 3.9.4 (available at https://www.python.org/downloads/). For additional details, please refer to the accompanying figure. The classification target was the resistance of *A. fumigatus* to the three azoles was. A comprehensive suite of ten machine learning classifiers was utilized in the model development process, as detailed in the accompanying table. These classifiers include logistic regression (LR), support vector classifiers (SVC, encompassing SVC RBF and SVC linear), decision trees (DT), ensemble learning methods (specifically Random Forest and AdaBoost), and XGBoost. The programming language employed for this endeavor was Python version 3.8.4. For logistic regression models incorporating regularization, three types were examined: L1 regularization (lasso regression), L2 regularization (ridge regression), and a hybrid approach combining of L1 and L2 regularization (elastic net).

Hyperparameters for penalized logistic regression models were optimized using grid search with five-fold cross-validation within the training set, using AUC as the scoring metric. For Lasso and Ridge logistic regression, the inverse regularization parameter C was tuned using the liblinear solver, whereas Elastic Net was tuned by jointly optimizing C and L1_ratio using the saga solver. The most frequently selected optimal parameter across repeated grid-search runs was used for subsequent model fitting. Tree-based and ensemble models, including Random Forest, AdaBoost, and XGBoost, were implemented with 100 estimators in the main performance analysis. The detailed hyperparameter grids and model settings are provided in Supplementary Table S4.

**Figure 5:**
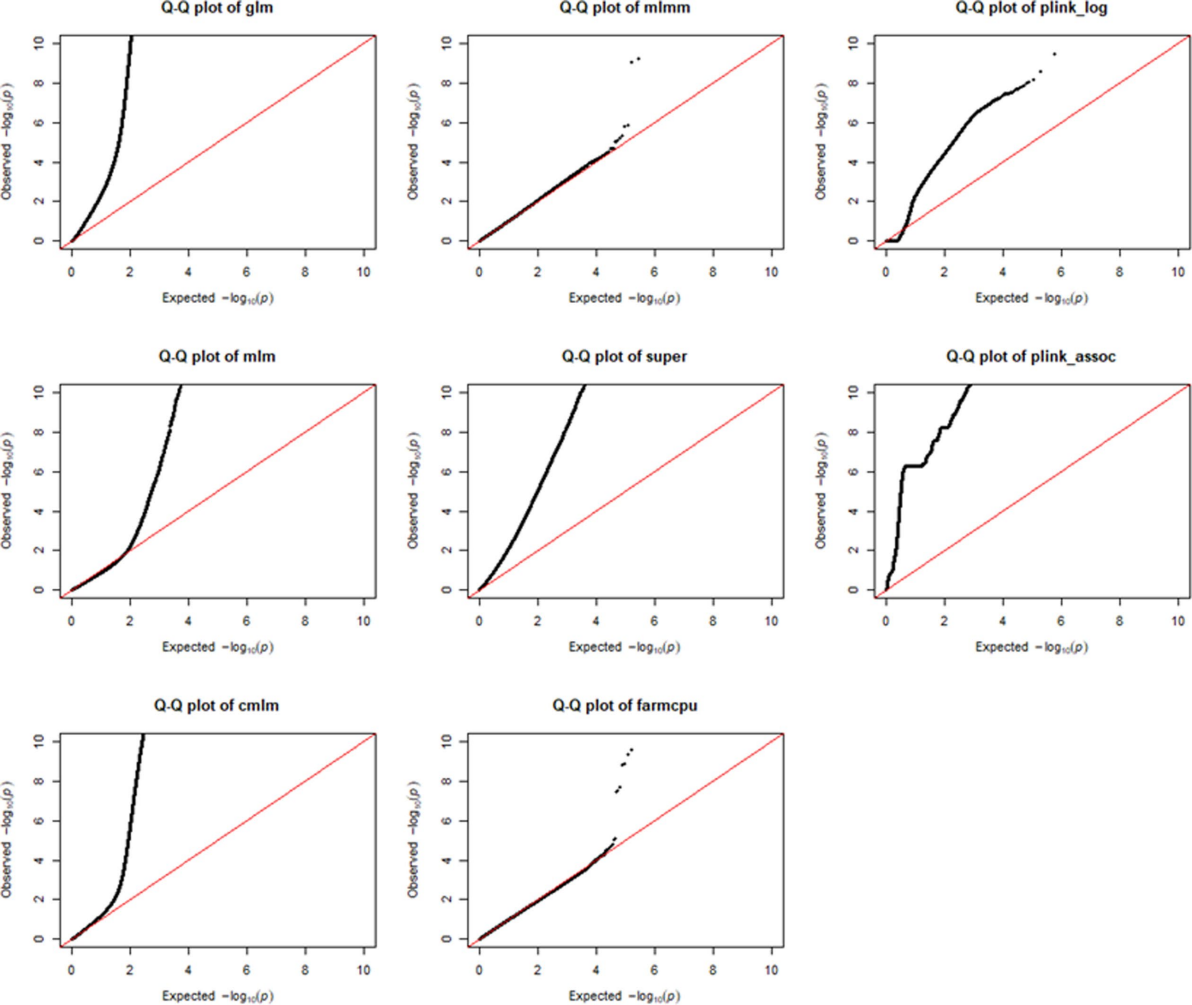
Predictive accuracy of three different azole phenotypes using various machine learning prediction methods.

**Figure 6:**
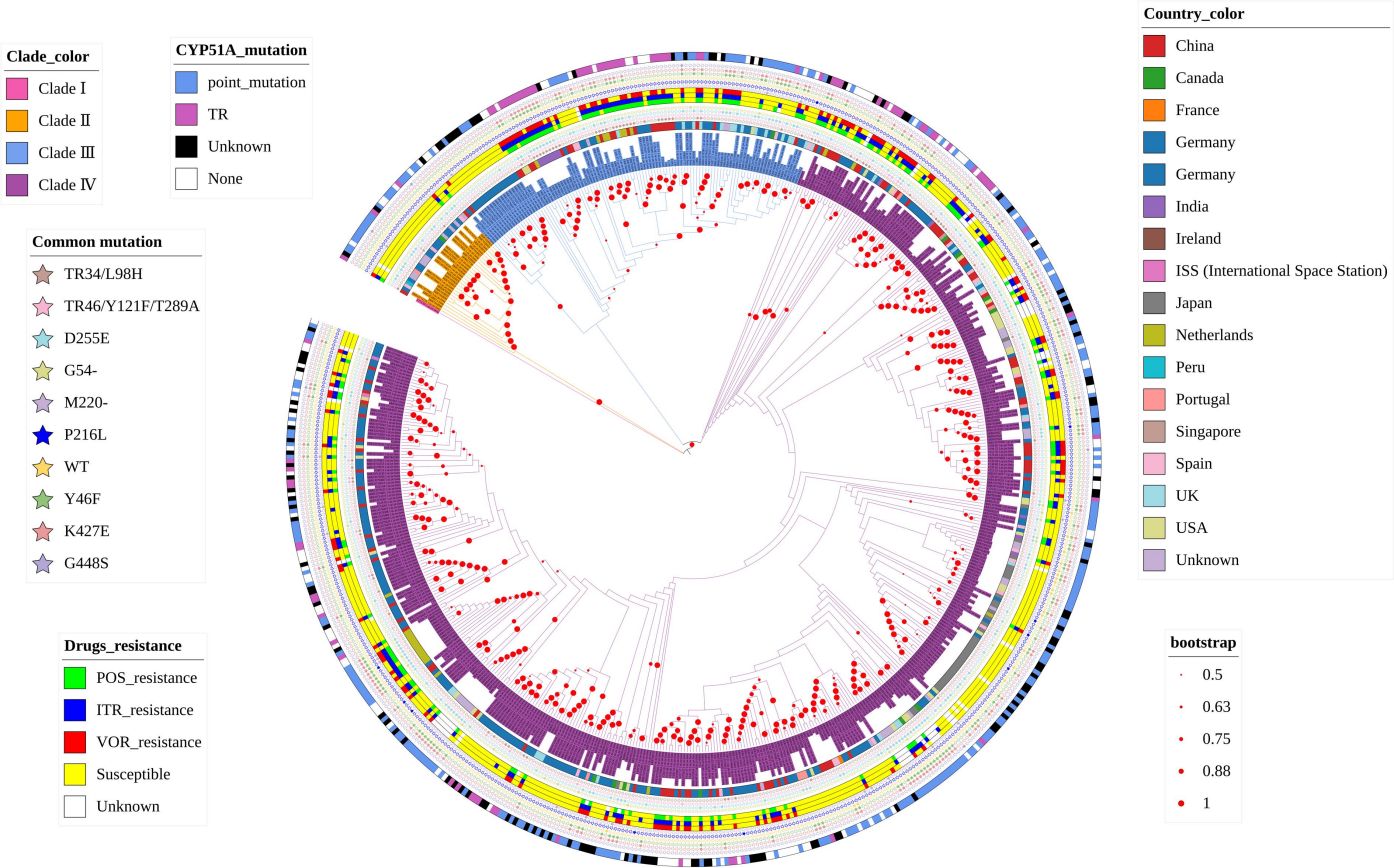
AUC values for three different azole phenotypes using diverse machine learning prediction methods.

Model performance was evaluated using 100 repeated random 80:20 train-test splits. In each repetition, 80% of the isolates were used as the training set and the remaining 20% were held out as the test set. Within the training set, five-fold cross-validation was used for hyperparameter tuning and RFECV-based feature selection where applicable. The optimized model was then refitted on the full training set and evaluated on the held-out test set. AUC was used as the primary performance metric, and accuracy (ACC) was also recorded. The AUC served as the primary parameter for evaluating model performance, with higher AUC values (approaching 1.0) indicating superior classifier performance. The prediction variance among models, observed over 100 iterations for three distinct azole phenotypes, is depicted in Figures 5, 6.

### 2.7 Feature selection process for penalized logistic regression models

The model is calibrated using the training dataset, with feature sparsity regulated through the adjustment of regularization parameters during the fitting process. In this study, a grid search methodology is employed to determine the optimal regularization parameters. Subsequently, the coefficients of all features are derived from the trained model and ranked in descending order based on their absolute values.

### 2.8 Recursive feature elimination with cross-validation

Given that most machine learning algorithms are primarily designed for classification tasks, and do not inherently include feature screening, we propose employing the recursive feature elimination (RFE) method to classify and predict the most significant loci. This approach aims to identify mutation loci associated with *A. fumigatus* resistance, thereby exploring loci linked to various azole resistances.

In this study, we employ the Python programming language to implement a RFE technique. Specifically, we employ the Recursive Feature Elimination with Cross-Validation (RFECV) class function from the Python scikit-learn (Pedregosa et al., 2011) library to facilitate recursive feature elimination with cross-validation. The “estimator” parameter specifies the type of model to be trained, while the “step” parameter dictates the number of features to be removed in each iteration. In this instance, the step size is set to 1, indicating a sequential elimination process. The model development is conducted using a 5-fold cross-validation (CV) approach. The training models are selected from the machine learning classifiers previously described, utilizing the dataset employed for modeling the forward machine learning approach. RFECV rankings were used to prioritize candidate resistance-associated loci.

## 3 Result

### 3.1 Evaluation of classification models

The machine-learning dataset consisted of 7,098 candidate SNP features derived from the merged GWAS-screened SNP set and literature-supported resistance loci. Drug-specific models were trained after excluding isolates with unknown phenotypes, resulting in 550 isolates for VOR, 537 for POS, and 549 for ITR. In this study, model performance was evaluated using 100 repeated random 80:20 train-test splits for each azole phenotype. Five-fold cross-validation was used within the training set for hyperparameter tuning and RFECV-based feature selection where applicable. Model performance across 100 repetitions was summarized as mean, standard deviation and visualized using box plots. Where appropriate, paired comparisons between top-performing models were performed using repeated-split AUC values. The resulting ACC and AUC distributions are shown in Figures 5, 6.

Because resistant and susceptible isolates were imbalanced for each azole phenotype, AUC was considered the primary performance metric, whereas ACC was interpreted as a threshold-dependent secondary metric. The decision tree approach exhibited the lowest average prediction accuracy among with a value of 79.8%. Additionally, its AUC value was found to be below 80%. In contrast, logistic regression with penalties demonstrated superior average prediction accuracy compared to the other six machine learning models. The AdaBoost model showed suboptimal prediction accuracy for ITR and POS, but performed relatively well for VOR. Both Random Forest and XGBoost displayed moderate prediction accuracy across all azoles. The average prediction accuracies for Lasso, ElasticNet, Ridge regression and SVM-rbf were 88.54, 55.56, 55.71, and 88.25%, respectively.

The subsequent analysis will focus on analyze the AUC values of each model. Although the AUC values of different azoles may vary across models, their performance remains largely consistent. Notably, the AUC values for the decision tree and AdaBoost models are predominantly below 90%. Moreover, logistic regression shows relatively consistent performance for ITR, but exhibits lower AUC values and greater variance for the other two drugs. The models exhibiting the highest AUC values were selected for further comparative analysis. Elastic Net and Ridge regression showed high mean AUC values nearing 95% across the three azoles. Random Forest, XGBoost, and SVM-based models also showed relatively high internal performance in repeated train-test evaluations. Models with unstable performance across azoles were not emphasized in subsequent biological interpretation, whereas models with consistently higher internal AUC values were used to prioritize candidate SNPs.

### 3.2 Mutation ranking

RFECV and penalized logistic regression were employed to rank candidate SNP features and construct predictive models for POS, ITR, and VOR resistance. Random Forest, XGBoost, and linear-kernel SVM showed relatively high internal performance and were therefore selected for RFECV feature ranking. This outcome is attributed to the inability to construct the SVM model with a polynomial kernel function for recursive feature screening. In this study, the Random Forest and XGBoost models, as along with the SVM method utilizing a linear kernel function, were employed for recursive feature elimination. Furthermore, logistic regression with elastic net and ridge regression penalties was employed to screen feature variables. These variables were subsequently ranked based on the absolute magnitude of their coefficients following the regression analysis. The results of mutation ranking derived from the aforementioned methodologies, are presented in the accompanying Supplementary Table S3.

The analysis identified and highlighted previously reported mutations, particularly in the cyp51B and cyp51A genes, which are currently recognized for their association with azole resistance. The ranking of specific loci as most significant in the RFECV led to the default output being ordered by the numerical size of the locus name. Consequently, the top 100 loci were selected for comprehensive analysis. In instances where the number of loci ranked first by a given method exceeded 100, all loci ranked first by that method were included. The resultant dataset revealed the extent of overlap between the top 100 loci and the established loci for each model in response to various azoles, as depicted in the accompanying table. Across the three azoles, “SCM000172.1_1781459” was repeatedly prioritized by multiple feature-ranking approaches. This SNP was annotated to AFUA_4G06890/cyp51A and corresponds to the L98H substitution, a component of the well-characterized TR34/L98H azole-resistance mechanism. Its repeated recovery supports the biological plausibility of the feature-ranking framework. The variable screening approach, employing logistic regression with an L2 penalty and an elastic net penalty, pinpointed “SCM000172.1_1781459” as the only locus among those known to be associated with drug resistance across all three azoles. This finding suggests a potential limitation of the method in effectively ranking feature importance.

Additionally, our results indicate that although the linear kernel function SVM model exhibits the highest number of overlap between prioritized loci and known resistance-associated loci, it frequently ranks a substantial number of loci equally at the top position. Because several methods produced tied rankings or model-specific feature priorities, we interpreted loci repeatedly identified across methods as more stable candidates rather than treating any single model as definitively superior.

Moreover, the performance of the various models varied when subjected to different azoles, potentially due to the multidrug resistance exhibited by *A. fumigatus*. Therefore, it is crucial to evaluate the number of overlapping resistances sites among different azoles when employing the XGBoost model, particularly focusing on loci ranked within the top 100. A significant overlap was observed in the resistance-associated loci of *A. fumigatus* for POS and ITR. In contrast, the overlap between loci associated with resistance to VOR and those associated with resistance to both POS and ITR was minimal. These findings suggest that the loci responsible for resistance to itraconazole in *A. fumigatus* frequently coincide with those responsible for resistance to POS, while having a limited impact on VOR resistance.

A comprehensive analysis and comparison of the two models, Random Forest and XGBoost, revealed that both models performed well, demonstrating high accuracy in model fitting. Furthermore, numerous recognized mutation sites were identified among the key mutation sites associated with *A. fumigatus* resistance, suggesting the feasibility of employing machine learning techniques to predict and rank mutations associated with resistance in *A. fumigatus*. Random Forest and XGBoost prioritized several known resistance-associated loci among the top-ranked features, supporting the utility of machine-learning approaches for generating candidate SNP lists. This finding fulfills the objective of enhanced detection with a limited number of input samples. The methodology outlined in this study has identified additional mutation sites beyond those previously recognized, necessitating further biological experiments to verify their actual association with triazole resistance.

## 4 Discussion

In this study, we identified the most appropriate GWAS model for analyzing drug resistance in *A. fumigatus* by utilizing the existing GWAS models, thereby integrating GWAS and ML to prioritize candidate SNPs associated with azole resistance in *A. fumigatus* and to explore potential overlap among azole-resistance signals. Methodologically, the hybrid framework addresses limitations of conventional GWAS by reducing false positives and enhancing predictive power, offering a scalable approach for fungal genomics. This framework also offers a methodological basis conducting similar GWAS studies on other fungi in the future. However, since the GWAS models employed in this study are pre-existing, the analysis results still contain a substantial number of false positives. In future experiments, we aim to develop novel GWAS models tailored to the specific characteristics of *A. fumigatus* to enhance the precision of our analyses.

Machine learning techniques hold significant promise for classifying strain resistance using WGS data and for analyzing high-dimensional datasets, which are crucial for predicting resistance-associated mutations. Our evaluation of model performance indicates substantial variability in the efficacy of machine learning classifiers when tested across different drugs. Several classifiers showed promising internal performance across the three azoles, particularly when evaluated by AUC. However, there were limitations in model optimization, as only a limited number of models underwent optimization during the classifier construction and parameter adjustment phases. Consequently, future research should focus on comprehensive model optimization through extensive experimentation and testing to achieve improved performance.

In this study, RFECV in conjunction with machine learning classifiers and regularized logistic models was employed to predict and rank mutations in *A. fumigatus* associated with triazole resistance. The RFECV process yielded varying results for mutation, rankings depending on the classifiers used. The recovery of several known resistance-associated loci supports the biological plausibility of the feature-ranking framework. Additional loci prioritized by the models should be regarded as candidate variants that require functional validation. Additionally, it was observed that among the loci associated with azole resistance in *A. fumigatus*, there is a frequent occurrence of resistance to both itraconazole and posaconazole, while the loci conferring resistance to voriconazole appear to be relatively independent of the aforementioned drugs. These results underscore the necessity for further investigation and verification of the specific mutation and drug resistance mechanisms in *A. fumigatus*.

In the context of machine learning, challenges such as overfitting, underfitting, noisy data, and in sufficient validation are prevalent. Therefore, incorporating all available variants and employing machine learning techniques to reduce dimensionality can improve model performance. Future research should prioritize the systematic validation of these mutations and conduct functional studies to elucidate their implications.

These findings provide a prioritized set of candidate loci for future mechanistic of *A. fumigatus* studies and may inform subsequent surveillance-oriented research, but clinical or therapeutic applications require further validation.

## Data Availability

The newly generated whole-genome sequencing data for Aspergillus fumigatus isolates have been deposited in the NCBI Sequence Read Archive under BioProject accession number PRJNA1480373. Publicly available sequencing data reused in this study are listed in Supplementary Table S1.

## Footnotes

1
https://itol.embl.de/

## Supplementary material

SUPPLEMENTARY TABLE S1 Additional information on a total of 590 *Aspergillus fumigatus* genomes.

SUPPLEMENTARY TABLE S2 Repeated random 80:20 train-test split results for each azole phenotype across different machine-learning models, with 100 repetitions.

SUPPLEMENTARY TABLE S3 The top 100 resistance loci associated with three azoles identified through recursive feature screening using various models.

SUPPLEMENTARY TABLE S4 Machine-learning model settings, hyperparameter grids, and validation procedures.
