# Supplementary material for: Genomic variant-driven prediction of azole resistance in Aspergillus fumigatus using GWAS and machine learning
Source: Front Microbiol. 2026 Jul 7;17:1891518. doi: 10.3389/fmicb.2026.1891518 (PMC13389983; doi:10.3389/fmicb.2026.1891518)
Supplement: SUPPLEMENTARY TABLE S4 — Machine-learning model settings, hyperparameter grids, and validation procedures. [file Table_4.DOCX]

**Table S4.** Machine-learning model settings, hyperparameter grids, and validation procedures.

| **Model / procedure** | **Implementation** | **Fixed settings** | **Hyperparameter grid / searched parameters** | **Tuning or selection strategy** | **Random-state setting** |
| --- | --- | --- | --- | --- | --- |
| Logistic regression | sklearn.linear_model.LogisticRegression | penalty = None; solver = lbfgs; max_iter = 1000 or 3000 where required | Not tuned | Used as the baseline classifier in repeated train-test evaluation | random_state = 0 where specified |
| Lasso logistic regression | sklearn.linear_model.LogisticRegression | penalty = l1; solver = liblinear | C = 0.01, 0.1, 1, 10, 100 | GridSearchCV with five-fold CV within the training set; modal optimal C across repeated searches was used for fitting | Not uniformly fixed for all train-test splits |
| Ridge logistic regression | sklearn.linear_model.LogisticRegression | penalty = l2; solver = liblinear | C = 0.01, 0.1, 1, 10, 100 | GridSearchCV with five-fold CV within the training set; modal optimal C across repeated searches was used for fitting | Not uniformly fixed for all train-test splits |
| Elastic Net logistic regression | sklearn.linear_model.LogisticRegression | penalty = elasticnet; solver = saga; max_iter = 500 or 1000 | C = 0.01, 0.1, 1, 10, 100; l1_ratio = 0 to 1 | GridSearchCV with five-fold CV within the training set; modal optimal C and l1_ratio across repeated searches were used for fitting | Not uniformly fixed for all train-test splits |
| Linear SVC | sklearn.svm.SVC | kernel = linear; probability = True; gamma = auto where used | C = 1 or 100 in different scripts | Main model evaluation and RFECV-based feature selection where applicable | Not uniformly fixed |
| RBF SVC | sklearn.svm.SVC | kernel = rbf; probability = True; gamma = auto where used | C = 1 or 100 in different scripts | Main model evaluation | Not uniformly fixed |
| Decision tree | sklearn.tree.DecisionTreeClassifier | Default parameters unless otherwise specified | Not tuned in the main analysis | Main model evaluation and RFECV-based feature selection where applicable | random_state = 0 where specified |
| Random Forest | sklearn.ensemble.RandomForestClassifier | Main analysis: n_estimators = 100 | Additional grid: n_estimators = 100, 200, 300, 500; max_depth = None, 10, 20, 30; max_features = sqrt, log2; min_samples_split = 2, 5, 10; min_samples_leaf = 1, 2, 4 | Main model evaluation; additional GridSearchCV with five-fold CV for Random Forest tuning | random_state = 0 |
| AdaBoost | sklearn.ensemble.AdaBoostClassifier | n_estimators = 100 | Not tuned in the main analysis | Main model evaluation and RFECV-based feature selection where applicable | random_state = 0 where specified |
| XGBoost | xgboost.XGBClassifier | n_estimators = 100 | Not tuned in the main analysis | Main model evaluation and RFECV-based feature selection where applicable | random_state = 0 where specified |
| RFECV feature selection | sklearn.feature_selection.RFECV | step = 1; cv = StratifiedKFold(5) | Number of retained features selected automatically | Recursive feature elimination with stratified five-fold CV | Depends on the estimator |
| Model evaluation | Custom Python scripts | 100 repeated random 80:20 train-test splits | Not applicable | Models were trained on 80% of isolates and evaluated on the held-out 20% test set in each repetition | Random split was not uniformly seeded |

Abbreviations: CV, cross-validation; RFECV, recursive feature elimination with cross-validation; SVC, support vector classifier. AUC and ACC were recorded for model evaluation in the main analysis. No primary resampling-based class-imbalance correction was applied in the main analysis. The random train-test split was not uniformly seeded unless random_state is explicitly stated.
